# 5,6-Dichloro-2-(2-fluoro­phen­yl)iso­indoline-1,3-dione

**DOI:** 10.1107/S1600536808010544

**Published:** 2008-04-23

**Authors:** Orhan Büyükgüngör, Mustafa Odabaşoğlu

**Affiliations:** a Department of Physics, Faculty of Arts and Sciences, Ondokuz Mayıs University, TR-55139 Kurupelit Samsun, Turkey; b Department of Chemistry, Faculty of Arts and Sciences, Ondokuz Mayıs University, TR-55139 Kurupelit Samsun, Turkey

## Abstract

The crystal structure of the title compound, C_14_H_6_Cl_2_FNO_2_, exhibits C—H⋯π and π–π inter­actions, which generate *C*(3) chains in the [100] direction. The π–π inter­action occurs between the aromatic rings of isoindoline units, with a centroid–centroid distance of 3.672 Å and an inter­planar separation of 3.528 Å. The isoindoline unit is planar and inclined at an angle of 58.63 (18)° to the substituent benzene ring. The F atom is disordered over two positions, with refined occupancies of 0.669 (3) and 0.331 (3).

## Related literature

For general background, see: Hall *et al.* (1987 ▶); Abdel-Hafez (2004 ▶); Sena *et al.* (2007 ▶). For related literature, see: Loudon (2002 ▶).
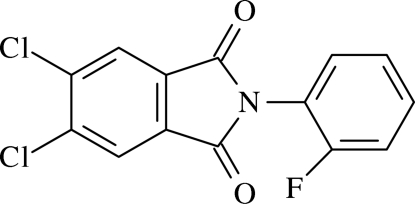


## Experimental

### 

#### Crystal data

C_14_H_6_Cl_2_FNO_2_
*M* *_r_* = 310.11
Orthorhombic, 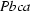

*a* = 8.0078 (3) Å
*b* = 27.3570 (9) Å
*c* = 11.5563 (5) Å
*V* = 2531.63 (17) Å^3^
*Z* = 8
Mo *K*α radiation
μ = 0.52 mm^−1^
*T* = 296 K
0.76 × 0.50 × 0.20 mm

#### Data collection

Stoe IPDSII diffractometer
Absorption correction: integration (*X-RED32*; Stoe & Cie, 2002 ▶) *T*_min_ = 0.703, *T*_max_ = 0.907
31181 measured reflections
2384 independent reflections
1956 reflections with *I* > 2σ(*I*)
*R*_int_ = 0.063

#### Refinement

*R*[*F* ^2^ > 2σ(*F* ^2^)] = 0.031
*wR*(*F* ^2^) = 0.085
*S* = 1.03
2384 reflections
192 parameters
H-atom parameters constrained
Δρ_max_ = 0.15 e Å^−3^
Δρ_min_ = −0.17 e Å^−3^

### 

Data collection: *X-AREA* (Stoe & Cie, 2002 ▶); cell refinement: *X-AREA*; data reduction: *X-RED32* (Stoe & Cie, 2002 ▶); program(s) used to solve structure: *SHELXS97* (Sheldrick, 2008 ▶); program(s) used to refine structure: *SHELXL97* (Sheldrick, 2008 ▶); molecular graphics: *ORTEP-3 for Windows* (Farrugia, 1997 ▶); software used to prepare material for publication: *WinGX* (Farrugia, 1999 ▶).

## Supplementary Material

Crystal structure: contains datablocks I, global. DOI: 10.1107/S1600536808010544/bt2693sup1.cif

Structure factors: contains datablocks I. DOI: 10.1107/S1600536808010544/bt2693Isup2.hkl

Additional supplementary materials:  crystallographic information; 3D view; checkCIF report

## Figures and Tables

**Table 1:** Hydrogen-bond geometry (Å, °)

*D*—H⋯*A*	*D*—H	H⋯*A*	*D*⋯*A*	*D*—H⋯*A*
C6—H6⋯*Cg*1^i^	0.93	2.99	3.844 (4)	153

Symmetry code: (i) 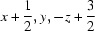. *Cg*1 is the centroid of atoms C2–C7.

**Fig. 1.**
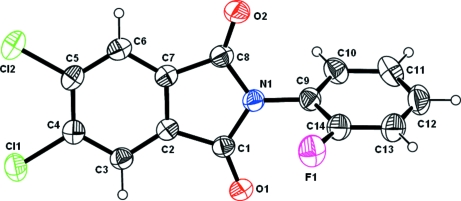
The molecular structure of the title molecule, with the atom-numbering scheme. Displacement ellipsoids are drawn at the 30% probability level. Only the higher occupied of the disordered sites is shown.

**Fig. 2.**
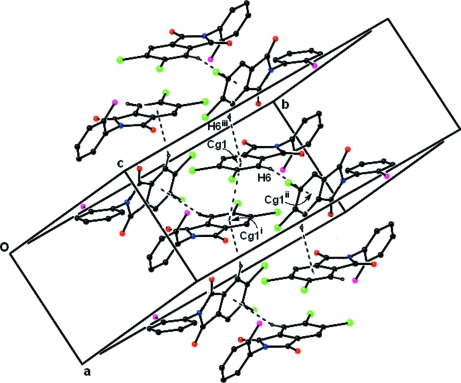
A partial packing diagram of (I), showing the formation of C(3) chain and π–π interactions. H atoms not involved in hydrogen bonds have been omitted for clarity. [Symmetry codes: (i) 1 - x,1 - y,1 - z; (ii) 1/2 + x, y, 3/2 - z; (iii) x - 1/2, y, 3/2 - z].

**Fig. 3.**
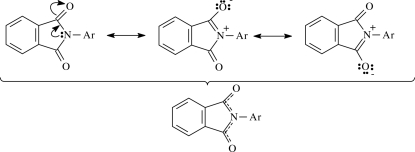
The sp2 hybridization of the adjacent carbon and the overlap of unshared electrons on nitrogen with π-electron system of carbonyl groups.

## supplementary crystallographic information

### Comment

Phthalimide derivatives have various biological activities (Hall *et al.*, 1987; 1987; Abdel-Hafez, 2004; Sena *et al.* 2007). In view of the importance of the *N*-arylphthalimides, we herein report the results of title compound 5,6-dichloro-2-(2-fluorophenyl)isoindoline-1,3-dione, (I).

The molecule of (I) is built up from a 5,6-dichlorophthalimide unit connected to a *o*-fluorophenyl group through an nitrogen atom (Fig. 1). The isoindoline ring (atoms N1/C1–C8) is almost planar the largest deviation from the mean plane being 0.027 (2) Å for atom C1. The dihedral angle between the fluorophenyl ring and the mean plane of the isoindoline part is 58.63 (18)°. In (I), the crystal packing is stabilized by C6—H6···π (Table 1) interactions. The C1—N1 and C8—N1 bonds are 1.406 (2) and 1.394 (2)Å, respectively. These C—N bond lengths are shorter than C—N single bond (C—N = 1.47 Å; Loudon, 2002). This reflects both the *sp*^2^ hybridization of the adjacent carbon and the overlap of unshared electrons on nitrogen with π-electron system of carbonyl groups (Fig. 3). The π–π interaction occurs between the aromatic rings (C2–C7) of isoindoline moieties at (*x*; *y*; *z*) and (1 - *x*; 1 - *y*; 1 - *z*) sites with the centroid-centroid distance of 3.672 Å and an interplanar separation of 3.528 Å.

### Experimental

A mixture of 4,5-dichlorophthalic acid (1.175 g, 0.005 mol) and 2-fluoroaniline (0.56 g, 0.005 mol) in DMF (1.5 ml) was heated at boiling temperature 15 min. The reaction mixture added in 50 ml e thanol (95%) and crystals of (I) suitable for X-ray analysis were obtained by slow evaporation of this mixture at room temperature (yield 80%).

### Refinement

The F atom is disordered over two *ortho* positions with refined occupancies of 0.669 (3) and 0.331 (3). H atoms were positioned geometrically, with C—H = 0.93 Å for aromatic H, and constrained to ride on their parent atoms with *U*_iso_(H) = 1.2*U*_eq_(C).

### Crystal data

**Table:**

C_14_H_6_Cl_2_FNO_2_	*F*_000_ = 1248
*M**_r_* = 310.11	*D*_x_ = 1.622 Mg m^−^^3^
Orthorhombic, *P**b**c**a*	Mo *K*α radiation λ = 0.71073 Å
Hall symbol: -P 2ac 2ab	Cell parameters from 31181 reflections
*a* = 8.0078 (3) Å	θ = 1.5–26.2º
*b* = 27.3570 (9) Å	µ = 0.52 mm^−^^1^
*c* = 11.5563 (5) Å	*T* = 296 K
*V* = 2531.63 (17) Å^3^	Prism, colorless
*Z* = 8	0.76 × 0.50 × 0.20 mm

### Data collection

**Table:**

Stoe IPDSII diffractometer	2384 independent reflections
Monochromator: plane graphite	1956 reflections with *I* > 2σ(*I*)
Detector resolution: 6.67 pixels mm^-1^	*R*_int_ = 0.063
*T* = 296 K	θ_max_ = 25.6º
ω scan rotation method	θ_min_ = 1.5º
Absorption correction: integration(X-RED32; Stoe & Cie, 2002)	*h* = −9→9
*T*_min_ = 0.703, *T*_max_ = 0.907	*k* = −33→33
31181 measured reflections	*l* = −14→14

### Refinement

**Table:**

Refinement on *F*^2^	Hydrogen site location: inferred from neighbouring sites
Least-squares matrix: full	H-atom parameters constrained
*R*[*F*^2^ > 2σ(*F*^2^)] = 0.031	*w* = 1/[σ^2^(*F*_o_^2^) + (0.0471*P*)^2^ + 0.3058*P*] where *P* = (*F*_o_^2^ + 2*F*_c_^2^)/3
*wR*(*F*^2^) = 0.085	(Δ/σ)_max_ = 0.001
*S* = 1.03	Δρ_max_ = 0.15 e Å^−^^3^
2384 reflections	Δρ_min_ = −0.17 e Å^−^^3^
192 parameters	Extinction correction: SHELXL97 (Sheldrick, 2008), Fc^*^=kFc[1+0.001xFc^2^λ^3^/sin(2θ)]^-1/4^
Primary atom site location: structure-invariant direct methods	Extinction coefficient: 0.0107 (9)
Secondary atom site location: difference Fourier map	

### Special details

**Table:**

Geometry. All e.s.d.'s (except the e.s.d. in the dihedral angle between two l.s. planes) are estimated using the full covariance matrix. The cell e.s.d.'s are taken into account individually in the estimation of e.s.d.'s in distances, angles and torsion angles; correlations between e.s.d.'s in cell parameters are only used when they are defined by crystal symmetry. An approximate (isotropic) treatment of cell e.s.d.'s is used for estimating e.s.d.'s involving l.s. planes.
Refinement. Refinement of *F*^2^ against ALL reflections. The weighted *R*-factor *wR* and goodness of fit *S* are based on *F*^2^, conventional *R*-factors *R* are based on *F*, with *F* set to zero for negative *F*^2^. The threshold expression of *F*^2^ > σ(*F*^2^) is used only for calculating *R*-factors(gt) *etc*. and is not relevant to the choice of reflections for refinement. *R*-factors based on *F*^2^ are statistically about twice as large as those based on *F*, and *R*- factors based on ALL data will be even larger.

### Fractional atomic coordinates and isotropic or equivalent isotropic displacement parameters (Å^2^)

**Table:**

	*x*	*y*	*z*	*U*_iso_*/*U*_eq_	Occ. (<1)
C1	0.28581 (19)	0.61572 (6)	0.53527 (14)	0.0525 (4)	
C2	0.30531 (19)	0.56310 (6)	0.56377 (13)	0.0503 (4)	
C3	0.2424 (2)	0.52248 (6)	0.50902 (14)	0.0548 (4)	
H3	0.1761	0.5254	0.4433	0.066*	
C4	0.2809 (2)	0.47693 (6)	0.55490 (15)	0.0554 (4)	
C5	0.3825 (2)	0.47286 (6)	0.65266 (15)	0.0578 (4)	
C6	0.4455 (2)	0.51427 (7)	0.70638 (15)	0.0585 (4)	
H6	0.5133	0.5118	0.7715	0.070*	
C7	0.40453 (19)	0.55905 (6)	0.66043 (14)	0.0516 (4)	
C8	0.4518 (2)	0.60897 (7)	0.69910 (14)	0.0569 (4)	
C9	0.3879 (2)	0.69277 (6)	0.62605 (15)	0.0557 (4)	
C10	0.3223 (3)	0.71816 (7)	0.71829 (17)	0.0702 (5)	
C11	0.3314 (3)	0.76849 (8)	0.7209 (2)	0.0850 (6)	
H11	0.2870	0.7857	0.7831	0.102*	
C12	0.4067 (3)	0.79318 (8)	0.6309 (2)	0.0867 (7)	
H12	0.4126	0.8271	0.6326	0.104*	
C13	0.4725 (3)	0.76831 (8)	0.5395 (2)	0.0867 (7)	
H13	0.5230	0.7851	0.4790	0.104*	
C14	0.4637 (2)	0.71824 (7)	0.53769 (17)	0.0687 (5)	
N1	0.37841 (17)	0.64082 (5)	0.61972 (11)	0.0540 (3)	
O1	0.20978 (16)	0.63434 (4)	0.45785 (10)	0.0670 (3)	
O2	0.53535 (19)	0.62065 (5)	0.78115 (12)	0.0813 (4)	
Cl1	0.20110 (7)	0.425557 (17)	0.48901 (4)	0.07471 (19)	
Cl2	0.43176 (8)	0.416515 (19)	0.70844 (5)	0.0847 (2)	
F1	0.5355 (3)	0.69169 (7)	0.45401 (16)	0.0918 (7)	0.669 (3)
F2	0.2785 (5)	0.68818 (15)	0.8105 (3)	0.0846 (14)	0.331 (3)
H10	0.2721	0.7019	0.7812	0.102*	0.669 (3)
H14	0.5077	0.7027	0.4724	0.102*	0.331 (3)

### Atomic displacement parameters (Å^2^)

**Table:**

	*U*^11^	*U*^22^	*U*^33^	*U*^12^	*U*^13^	*U*^23^
C1	0.0502 (9)	0.0555 (9)	0.0519 (8)	0.0034 (7)	−0.0010 (7)	0.0013 (7)
C2	0.0466 (8)	0.0536 (9)	0.0508 (8)	0.0033 (7)	0.0030 (7)	0.0024 (7)
C3	0.0548 (9)	0.0580 (9)	0.0517 (9)	0.0044 (7)	−0.0005 (7)	0.0001 (7)
C4	0.0535 (9)	0.0547 (9)	0.0579 (9)	0.0009 (7)	0.0110 (8)	−0.0015 (7)
C5	0.0549 (9)	0.0571 (10)	0.0613 (9)	0.0070 (7)	0.0107 (8)	0.0102 (8)
C6	0.0530 (9)	0.0664 (11)	0.0559 (9)	0.0022 (8)	−0.0015 (7)	0.0100 (8)
C7	0.0451 (8)	0.0571 (9)	0.0527 (8)	0.0007 (7)	0.0012 (7)	0.0051 (7)
C8	0.0490 (9)	0.0633 (11)	0.0583 (9)	−0.0074 (8)	−0.0035 (8)	0.0073 (8)
C9	0.0495 (9)	0.0536 (9)	0.0642 (10)	−0.0071 (7)	−0.0033 (8)	0.0033 (8)
C10	0.0716 (12)	0.0697 (12)	0.0694 (12)	−0.0167 (10)	0.0057 (10)	−0.0074 (9)
C11	0.0756 (14)	0.0754 (13)	0.1040 (17)	−0.0095 (11)	0.0001 (12)	−0.0284 (12)
C12	0.0862 (15)	0.0553 (11)	0.1186 (19)	−0.0149 (10)	−0.0175 (14)	0.0007 (12)
C13	0.0981 (17)	0.0699 (13)	0.0921 (15)	−0.0261 (12)	−0.0062 (13)	0.0163 (11)
C14	0.0676 (12)	0.0668 (11)	0.0717 (11)	−0.0108 (9)	0.0053 (10)	0.0052 (9)
N1	0.0530 (8)	0.0533 (7)	0.0556 (7)	−0.0044 (6)	−0.0038 (6)	0.0048 (6)
O1	0.0792 (9)	0.0590 (7)	0.0629 (7)	0.0081 (6)	−0.0172 (6)	0.0035 (5)
O2	0.0848 (10)	0.0764 (9)	0.0826 (9)	−0.0197 (7)	−0.0339 (8)	0.0115 (7)
Cl1	0.0859 (4)	0.0561 (3)	0.0822 (3)	−0.0074 (2)	0.0026 (3)	−0.0062 (2)
Cl2	0.0989 (4)	0.0610 (3)	0.0943 (4)	0.0118 (3)	−0.0031 (3)	0.0214 (2)
F1	0.1037 (15)	0.0859 (13)	0.0857 (12)	−0.0125 (10)	0.0384 (11)	0.0034 (10)
F2	0.086 (3)	0.095 (3)	0.073 (2)	−0.018 (2)	0.0152 (18)	−0.0176 (18)

### Geometric parameters (Å, °)

**Table:**

C1—O1	1.1961 (19)	C9—C10	1.376 (3)
C1—N1	1.405 (2)	C9—C14	1.377 (2)
C1—C2	1.485 (2)	C9—N1	1.425 (2)
C2—C3	1.374 (2)	C10—C11	1.379 (3)
C2—C7	1.375 (2)	C10—F2	1.390 (4)
C3—C4	1.389 (2)	C10—H10	0.942 (2)
C3—H3	0.9300	C11—C12	1.379 (3)
C4—C5	1.396 (3)	C11—H11	0.9300
C4—Cl1	1.7213 (17)	C12—C13	1.362 (3)
C5—C6	1.387 (3)	C12—H12	0.9300
C5—Cl2	1.7169 (17)	C13—C14	1.372 (3)
C6—C7	1.375 (2)	C13—H13	0.9300
C6—H6	0.9300	C14—F1	1.339 (3)
C7—C8	1.486 (2)	C14—H14	0.935 (2)
C8—O2	1.204 (2)	F1—H14	0.4311 (19)
C8—N1	1.395 (2)	F2—H10	0.509 (4)
			
O1—C1—N1	125.46 (15)	C10—C9—N1	121.51 (15)
O1—C1—C2	129.22 (15)	C14—C9—N1	119.33 (16)
N1—C1—C2	105.32 (13)	C9—C10—C11	120.04 (19)
C3—C2—C7	121.37 (15)	C9—C10—F2	113.1 (2)
C3—C2—C1	130.02 (15)	C11—C10—F2	125.8 (2)
C7—C2—C1	108.60 (14)	C9—C10—H10	121.51 (19)
C2—C3—C4	117.92 (16)	C11—C10—H10	118.4 (2)
C2—C3—H3	121.0	C10—C11—C12	119.7 (2)
C4—C3—H3	121.0	C10—C11—H11	120.1
C3—C4—C5	120.63 (16)	C12—C11—H11	120.1
C3—C4—Cl1	118.77 (14)	C13—C12—C11	120.6 (2)
C5—C4—Cl1	120.61 (13)	C13—C12—H12	119.7
C6—C5—C4	120.61 (15)	C11—C12—H12	119.7
C6—C5—Cl2	118.79 (14)	C12—C13—C14	119.4 (2)
C4—C5—Cl2	120.60 (14)	C12—C13—H13	120.3
C7—C6—C5	117.91 (16)	C14—C13—H13	120.3
C7—C6—H6	121.0	F1—C14—C13	122.1 (2)
C5—C6—H6	121.0	F1—C14—C9	116.75 (17)
C6—C7—C2	121.55 (16)	C13—C14—C9	121.1 (2)
C6—C7—C8	129.94 (15)	C13—C14—H14	116.5 (2)
C2—C7—C8	108.50 (14)	C9—C14—H14	122.33 (19)
O2—C8—N1	125.90 (17)	C8—N1—C1	111.95 (13)
O2—C8—C7	128.49 (16)	C8—N1—C9	124.52 (14)
N1—C8—C7	105.61 (14)	C1—N1—C9	123.45 (14)
C10—C9—C14	119.16 (17)		
			
O1—C1—C2—C3	0.3 (3)	N1—C9—C10—C11	−179.04 (18)
N1—C1—C2—C3	−179.36 (16)	C14—C9—C10—F2	−168.5 (2)
O1—C1—C2—C7	179.79 (17)	N1—C9—C10—F2	11.8 (3)
N1—C1—C2—C7	0.13 (17)	C9—C10—C11—C12	−0.1 (3)
C7—C2—C3—C4	0.5 (2)	F2—C10—C11—C12	167.6 (3)
C1—C2—C3—C4	179.97 (15)	C10—C11—C12—C13	−0.2 (4)
C2—C3—C4—C5	−0.9 (2)	C11—C12—C13—C14	−0.1 (4)
C2—C3—C4—Cl1	179.09 (12)	C12—C13—C14—F1	−175.6 (2)
C3—C4—C5—C6	0.6 (3)	C12—C13—C14—C9	0.7 (3)
Cl1—C4—C5—C6	−179.39 (13)	C10—C9—C14—F1	175.55 (19)
C3—C4—C5—Cl2	−179.33 (13)	N1—C9—C14—F1	−4.7 (3)
Cl1—C4—C5—Cl2	0.7 (2)	C10—C9—C14—C13	−1.0 (3)
C4—C5—C6—C7	0.1 (2)	N1—C9—C14—C13	178.76 (19)
Cl2—C5—C6—C7	−179.98 (12)	O2—C8—N1—C1	−178.75 (17)
C5—C6—C7—C2	−0.5 (2)	C7—C8—N1—C1	1.21 (18)
C5—C6—C7—C8	179.55 (16)	O2—C8—N1—C9	−1.9 (3)
C3—C2—C7—C6	0.2 (2)	C7—C8—N1—C9	178.08 (14)
C1—C2—C7—C6	−179.39 (14)	O1—C1—N1—C8	179.45 (16)
C3—C2—C7—C8	−179.87 (15)	C2—C1—N1—C8	−0.86 (17)
C1—C2—C7—C8	0.59 (18)	O1—C1—N1—C9	2.5 (3)
C6—C7—C8—O2	−1.2 (3)	C2—C1—N1—C9	−177.77 (14)
C2—C7—C8—O2	178.87 (17)	C10—C9—N1—C8	−62.0 (2)
C6—C7—C8—N1	178.88 (16)	C14—C9—N1—C8	118.29 (19)
C2—C7—C8—N1	−1.10 (18)	C10—C9—N1—C1	114.5 (2)
C14—C9—C10—C11	0.7 (3)	C14—C9—N1—C1	−65.2 (2)

### Hydrogen-bond geometry (Å, °)

**Table:**

*D*—H···*A*	*D*—H	H···*A*	*D*···*A*	*D*—H···*A*
C6—H6···Cg1^i^	0.93	2.99	3.844 (4)	153

Symmetry codes: (i) *x*+1/2, *y*, −*z*+3/2.
